# Trinucleotide mRNA Cap Analogue *N*6-Benzylated at the Site of Posttranscriptional ^m6^A_m_ Mark Facilitates mRNA Purification and Confers Superior Translational Properties In Vitro and In Vivo

**DOI:** 10.1021/jacs.3c12629

**Published:** 2024-03-05

**Authors:** Marcin Warminski, Edyta Trepkowska, Miroslaw Smietanski, Pawel J. Sikorski, Marek R. Baranowski, Marcelina Bednarczyk, Hanna Kedzierska, Bartosz Majewski, Adam Mamot, Diana Papiernik, Agnieszka Popielec, Remigiusz A. Serwa, Brittany A. Shimanski, Piotr Sklepkiewicz, Marta Sklucka, Olga Sokolowska, Tomasz Spiewla, Diana Toczydlowska-Socha, Zofia Warminska, Karol Wolosewicz, Joanna Zuberek, Jeffrey S. Mugridge, Dominika Nowis, Jakub Golab, Jacek Jemielity, Joanna Kowalska

**Affiliations:** † Division of Biophysics, Institute of Experimental Physics, Faculty of Physics, University of Warsaw, 02-089 Warsaw, Poland; ‡ Centre of New Technologies, University of Warsaw, 02-089 Warsaw, Poland; § Explorna Therapeutics sp. z o.o. Zwirki i Wigury 93, 02-089 Warsaw, Poland; ∥ Laboratory of Epitranscriptomics, Department of Environmental Microbiology and Biotechnology, Institute of Microbiology, Faculty of Biology, Biological and Chemical Research Centre, University of Warsaw, 02-089 Warsaw, Poland; ⊥ Department of Chemistry & Biochemistry, University of Delaware, Newark, Delaware 19716, United States; # Laboratory of Experimental Medicine, Faculty of Medicine, Medical University of Warsaw, 02-097 Warsaw, Poland; ∇ Proteomics Core Facility, IMol Polish Academy of Sciences, 02-247 Warsaw, Poland

## Abstract

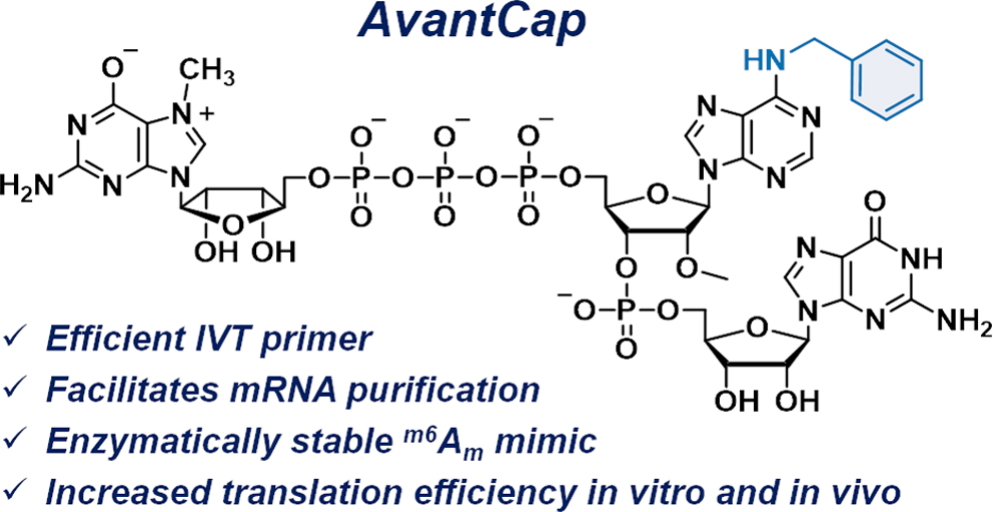

Eukaryotic mRNAs undergo cotranscriptional 5′-end modification with a 7-methylguanosine cap. In higher eukaryotes, the cap carries additional methylations, such as ^m6^A_m_—a common epitranscriptomic mark unique to the mRNA 5′-end. This modification is regulated by the Pcif1 methyltransferase and the FTO demethylase, but its biological function is still unknown. Here, we designed and synthesized a trinucleotide FTO-resistant *N*6-benzyl analogue of the ^m6^A_m_-cap–m^7^Gppp^Bn6^A_m_pG (termed *AvantCap*) and incorporated it into mRNA using T7 polymerase. mRNAs carrying ^Bn6^A_m_ showed several advantages over typical capped transcripts. The ^Bn6^A_m_ moiety was shown to act as a reversed-phase high-performance liquid chromatography (RP-HPLC) purification handle, allowing the separation of capped and uncapped RNA species, and to produce transcripts with lower dsRNA content than reference caps. In some cultured cells, ^Bn6^A_m_ mRNAs provided higher protein yields than mRNAs carrying A_m_ or ^m6^A_m_, although the effect was cell-line-dependent. m^7^Gppp^Bn6^A_m_pG-capped mRNAs encoding reporter proteins administered intravenously to mice provided up to 6-fold higher protein outputs than reference mRNAs, while mRNAs encoding tumor antigens showed superior activity in therapeutic settings as anticancer vaccines. The biochemical characterization suggests several phenomena potentially underlying the biological properties of *AvantCap*: (i) reduced propensity for unspecific interactions, (ii) involvement in alternative translation initiation, and (iii) subtle differences in mRNA impurity profiles or a combination of these effects. *AvantCapped-*mRNAs bearing the ^Bn6^A_m_ may pave the way for more potent mRNA-based vaccines and therapeutics and serve as molecular tools to unravel the role of ^m6^A_m_ in mRNA.

## Introduction

**Figure 1:**
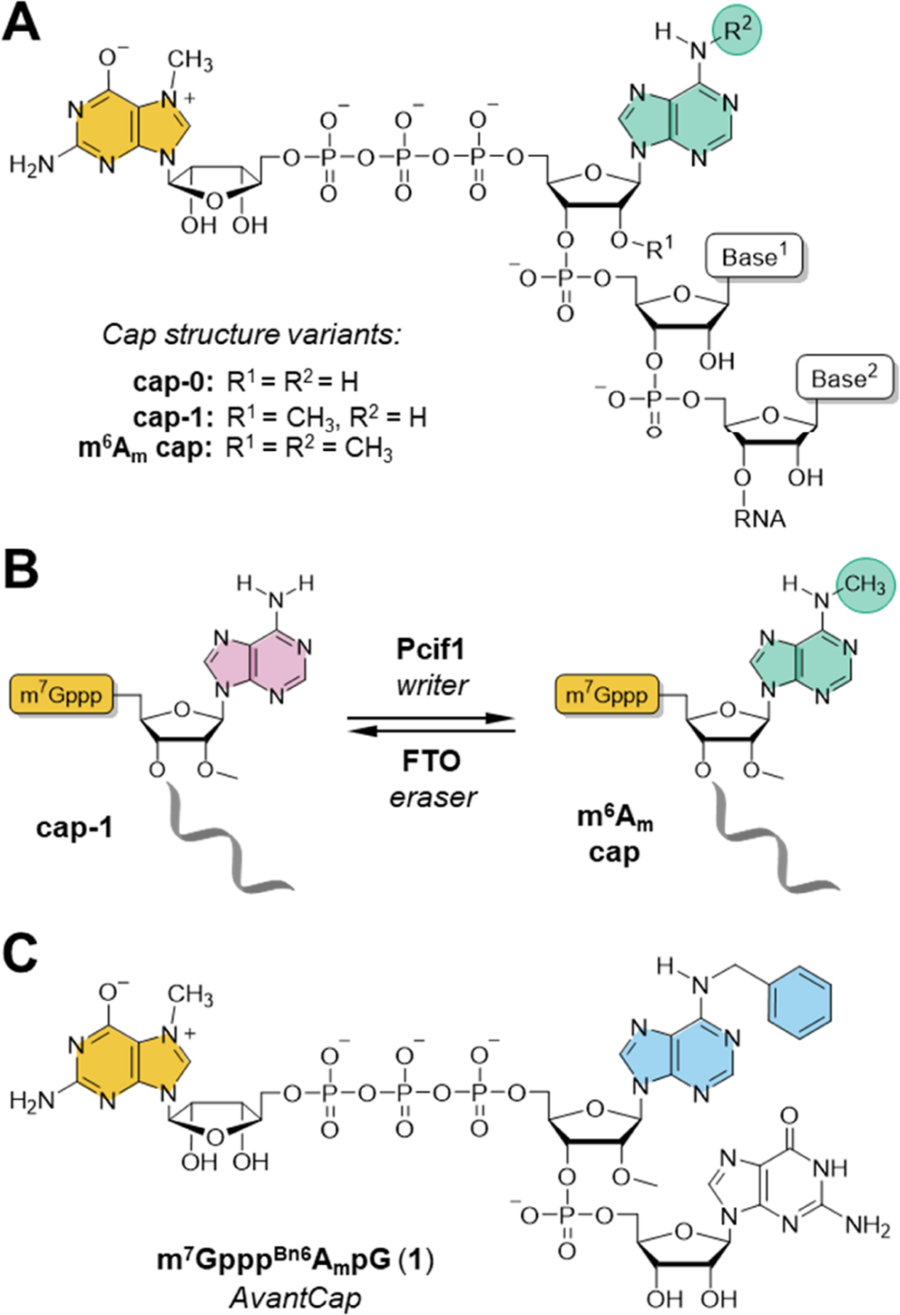
mRNA 5′ cap structure. (A) Natural variants of cap structures carrying adenosine adjacent to the 5′ cap; (B) dynamic regulation of ^m6^A_m_ presence in the cell; and (C) structure of m^7^Gppp^Bn6^A_m_pG (*AvantCap*).

Eukaryotic messenger RNAs (mRNAs) carry genetic information from the nucleus to the cytoplasm and serve as templates for protein biosynthesis in cells. As relatively short-lived and labile molecules, they are dynamically regulated on multiple levels, which play a major role in controlling gene expression. Chemical modifications are utilized by both nature and researchers to modulate the biological properties of mRNA. One of the earliest discovered natural modifications of eukaryotic mRNA is the 7-methylguanosine 5′ cap, which in humans is accompanied by additional methylations at the 2′-O position of the first one or two transcribed nucleotides (Figure 1A).^1^ Although not yet fully understood, these methylations play a vital role as epigenetic marks by which the cell distinguishes between its own and foreign mRNAs during viral infection.^2,3^ Analogous modifications are also introduced into exogenously delivered mRNA vaccines and therapeutics to make them resemble endogenous mRNA as much as possible.^4^ If adenosine is present as the first transcribed nucleotide (FTN) in mammalian mRNA, it can be additionally methylated at the *N*6-position to produce *N*6,2′-*O*-dimethyladenosine (^m6^A_m_).^5^ The methylation of adenine at the *N*6-position is a general regulatory mechanism in mRNA,^6^ but the biological effects of the ^m^^6^A presence strongly depend on its position in the mRNA body and the sequence context.^7^ The ^m6^A_m_ in mRNA is only found at the 5′ end (as FTN) and has an as-yet-unclear biological function. It may serve as a basis for dynamic translation regulation mechanisms, relying on the addition and removal of the methyl group by ^m^^6^A writers and erasers.^8,9^*N*-Methyltransferase Pcif1/CAPAM has been the only so far identified mRNA cap-specific ^m6^A writer,^10−14^ while FTO has been identified as the ^m6^A eraser for both internal ^m6^A and ^m6^A_m_ within the 5′ cap (Figure 1B).^15,16^ However, the biological effects of ^m^^6^A_m_ and underlying molecular mechanisms are still under debate since no cap-specific ^m6^A_m_ reader has been identified. Interestingly, the *N*6-methylation of adenosine as FTN is common in all types of mammalian cells and has been found to be relatively abundant in several tissues: e.g., in mice, the fraction of *N*6*-*methylated 5′ terminal A reaches around 70% in the liver, 90% in the heart, and 94% in the brain.^17,18^ The large differences in the ^m6^A_m_ content observed in transcripts from different genes also support this modification’s putative regulatory role.^19,20^

Over the past few years, the molecular effect of the ^m6^A_m_ modification on mRNA properties has been the subject of a lively scientific debate, with some conflicting data often resulting from the difficulty of separating the effects of 5′ cap-adjacent ^m6^A_m_ and internal ^m6^A modifications. Most of the researchers agree that the level of cap-adjacent ^m6^A_m_ positively correlates with the mRNA translation rate,^9^ although that effect seems to be cell-line or tissue-dependent.^2,21^ Direct comparison of bicistronic mRNAs capped with A_m_ or ^m6^A_m_ caps and containing IRES suggests that *N*6-methylation suppresses cap-dependent translation in rabbit reticulocyte lysate (RRL).^13^ To elucidate the biological role of ^m6^A_m_ mark in a more complex system, Akichika et al., Boulias et al., and Sendinc et al. independently studied the PCIF1 knockout cell lines.^10,11,13^ Under normal conditions, no difference in growth was observed between PCIF1 knockout and wild-type cells, while under oxidative stress, PCIF1-deficient cells showed defective growth,^10^ which suggests that ^m6^A_m_ is important for survival under stress conditions. In line with this observation, Sun et al. reported elevated *N*6-methylation of the 5′-cap under heat shock and hypoxia conditions, particularly within the mRNAs coding for proteins engaged in stress-response mechanisms.^20^ Gene ontology analysis of mRNAs isolated from mouse liver revealed that ^m6^A_m_ is clearly enriched in transcripts associated with mitochondrion and metabolic processes upon high-fat diet stress, linking the FTO activity with dynamic regulation of obesity.^22^ Studies in vivo showed that mice with mutations within the PCIF1 gene display a reduced body weight, but their viability and fertility were unaffected.^23^ A recent report has shown that *N*6-methylation of adenosine within the 5′ cap of viral mRNA attenuates the interferon-β-mediated suppression of viral infection,^24^ which suggests that this methylation may play a similar immunoregulatory role as 2′-*O*-methylations at the mRNA 5′ end.^2^ The methyltransferase activity of PCIF1 has also been shown to suppress HIV replication by enhancing the stability of host ^m6^A_m_-modified transcripts, which is circumvented by the viral protein-mediated PCIF1 degradation.^25^ Other studies linked PCIF1 methyltransferase activity with susceptibility to SARS-CoV-2 and other coronavirus infections,^26^ as well as with the response to transforming growth factor β (TGF-β) and to anti-PD-1 therapy in colorectal cancer cells.^27^

The ^m6^A_m_ mark can be incorporated into in vitro-transcribed (IVT) mRNA either by enzymatic posttranscriptional methylation^23,28^ or by cotranscriptional capping with the properly designed priming nucleotide.^15,20,21,29^ To enable more insight into the influence of ^m6^A_m_ on the properties of mRNA in different biological settings, we have previously used a transcription-priming trinucleotide cap analogue m^7^Gppp^m6^A_m_pG to evaluate translational properties of IVT mRNAs containing 5′ terminal ^m6^A_m_ in different cell lines.^21^ The presence of ^m6^A_m_ did not alter the translation of reporter mRNA in mouse fibroblasts (3T3-L1) but increased translation efficiency in human cancer (HeLa) and mouse dendritic (JAWSII) cells (compared to A_m_). The observed translation upregulation in specific cells, particularly in dendritic cells responsible for the generation of adaptive immunity, makes ^m6^A_m_ an exciting candidate for improving the dynamically developing mRNA vaccine field.^30^ However, further studies are needed to fully understand the biological role of this modification and harness its potential.

Synthetic modifications of mRNA 5′ cap have already proven to be an effective way to modulate translation efficiency of exogenously delivered transcripts.^31,32^ However, unnatural modifications of the first transcribed nucleotide have been rarely explored so far.^19,28^ This is mostly because the typical protocols for the preparation of capped mRNA utilize dinucleotide cap analogues, which are not compatible with the majority of modifications at the FTN position.^33^ However, recently developed tri- and tetranucleotide capping reagents overcome these limitations.^2,21,34^ Here, we aimed to develop a capping reagent introducing an ^m6^A_m_ mimic at the mRNA 5′ cap that would potentially retain the majority of its properties but was resistant to demethylation by FTO. We envisaged that at the molecular level, the methyl group can either stabilize complexes with proteins by hydrophobic interactions or destabilize them by steric hindrance. We hypothesized that both effects can be potentially enhanced by replacing the ^m6^A_m_ methyl group with a more bulky substituent such as benzyl, which has shown previously to be a good methyl mimic in terms of mRNA cap–protein interactions.^35^ Consequently, by replacing the *N*6-methyl group with benzyl in the m^7^Gppp^m6^A_m_pG cap structure, we developed a novel type of mRNA capping reagent–m^7^Gppp^Bn6^A_m_pG (Figure 1C). After careful evaluation of m^7^Gppp^Bn6^A_m_pG in vitro, in cultured cells, and in vivo in mouse models, aided by biochemical and proteomic experiments, we found that ^Bn6^A_m_ may indeed act as an FTO-resistant ^m6^A_m_ mimic that enhances the translational potential of IVT mRNA. Therefore, m^7^Gppp^Bn6^A_m_pG, which we termed *AvantCap*, is a highly promising reagent for the modification of IVT mRNA with high potential to reveal the biological nuances of ^m6^A_m_ functions as well as for therapeutic mRNA applications.

## Results

### Chemical Synthesis of AvantCap (**1**): An mRNA Cap Analogue Containing *N*6-Benzyl-2′-*O*-methyladenosine (^Bn6^A_m_)

**Scheme 1:**
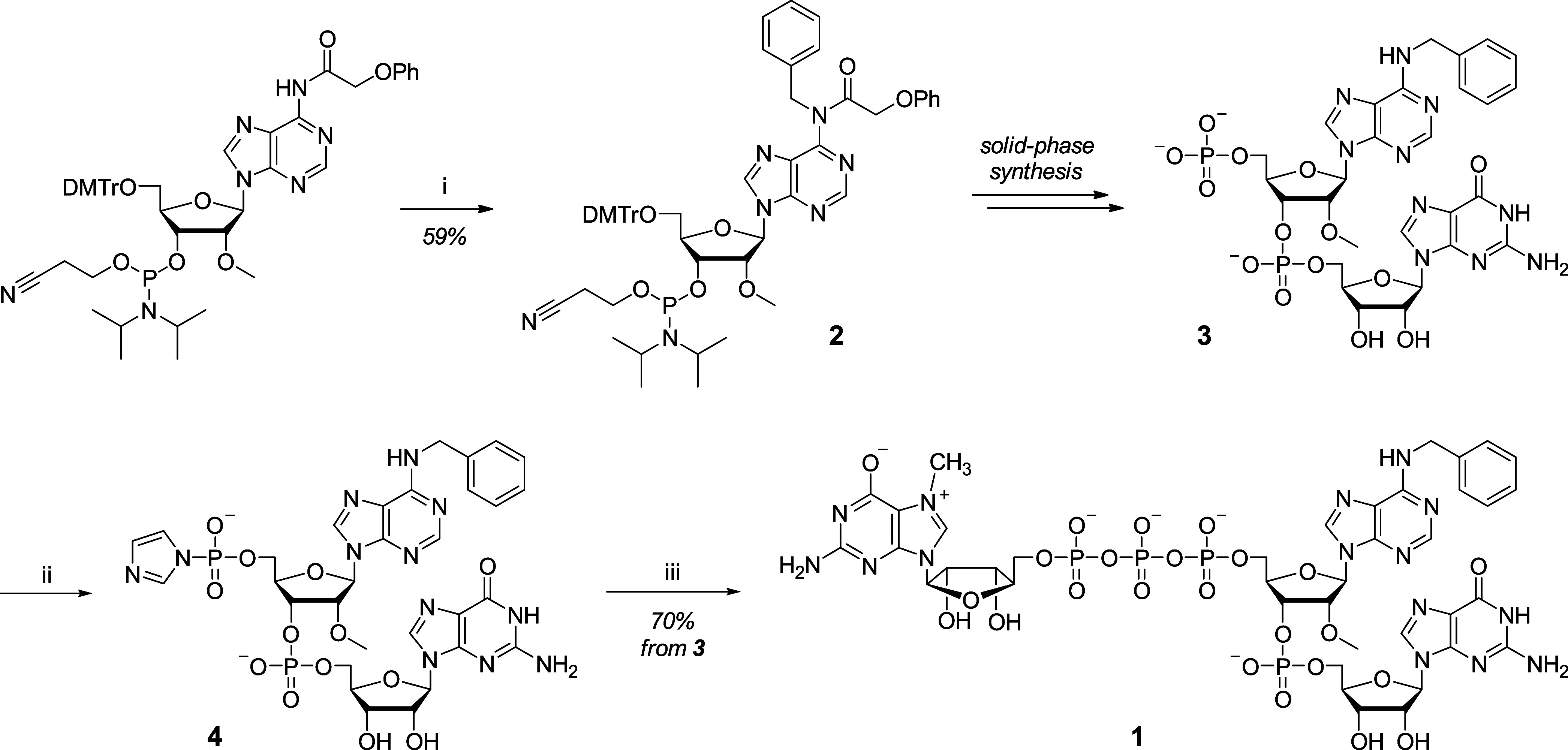
Chemical Synthesis of m^7^Gppp^Bn6^A_m_pG (**1**) Reaction conditions: (i) benzyl bromide, tetrabutylammonium bromide, 1 M NaOH_aq_, CH_2_Cl_2_; (ii) imidazole, 2,2′-dithiodipyridine, triphenylphosphine, triethylamine, DMF; and (iii) *N*7-methylguanosine 5′-diphosphate (m^7^GDP), ZnCl_2_, DMSO.

The synthetic pathway leading to trinucleotide cap analogue **1** included the solid-phase synthesis of dinucleotide 5′-monophosphate **3**, followed by its activation into *P*-imidazolide **4** and ZnCl_2_-mediated coupling reaction with 7-methylguanosine 5′-diphosphate (m^7^GDP) in solution (Scheme 1). The *N*6-benzyladenosine phosphoramidite for solid-phase synthesis was prepared by one-step alkylation of commercially available *N*6-phenoxyacetyl-2′-*O*-methyladenosine phosphoramidite in the presence of a base and phase-transfer catalyst.^17,36^ The dinucleotide 5′-phosphate **3** was cleaved from the solid support, deprotected using standard protocols, and then isolated by ion-exchange chromatography on DEAE Sephadex to give a triethylammonium salt suitable for further activation. The *P*-imidazolide **4** was prepared as described previously for mononucleotides,^37^ precipitated as a sodium salt and reacted with m^7^GDP in the presence of excess ZnCl_2_ to give trinucleotide cap analogue **1**. Activation of dinucleotide **3** instead of m^7^GDP appeared to be more efficient, particularly at larger scales (>50 μmol), and allowed to reduce the coupling time from 24 to 48 h to ca. 2 h. Compound **4** was relatively stable and was stored at −20–4 °C for several months without signs of decomposition. The final product **1** was isolated by ion-exchange chromatography and additionally purified by reversed-phase high-performance liquid chromatography (RP-HPLC) to give ammonium salts of **1** in good yield (70% starting from **3**). A typical solid-phase synthesis at a 200 μmol scale required 1.2–1.5 equiv of ^Bn6^A_m_ phosphoramidite and yielded ca. 140 μmol of **3**. We were able to upscale the procedure to obtain 3.15 g (ca. 2.5 mmol) of cap analogue **1** starting from an equivalent of 5 mmol of solid-supported guanosine and 10.3 g (10.2 mmol) of 2′-*O-*methyladenosine phosphoramidite.

### Characterization of *AvantCap*

#### AvantCap Initiates Transcription by T7 Polymerase to Produce m^7^Gppp^Bn6^A_m_pG-Capped RNA and Facilitates mRNA Purification by HPLC via Hydrophobic Effect

**Figure 2:**
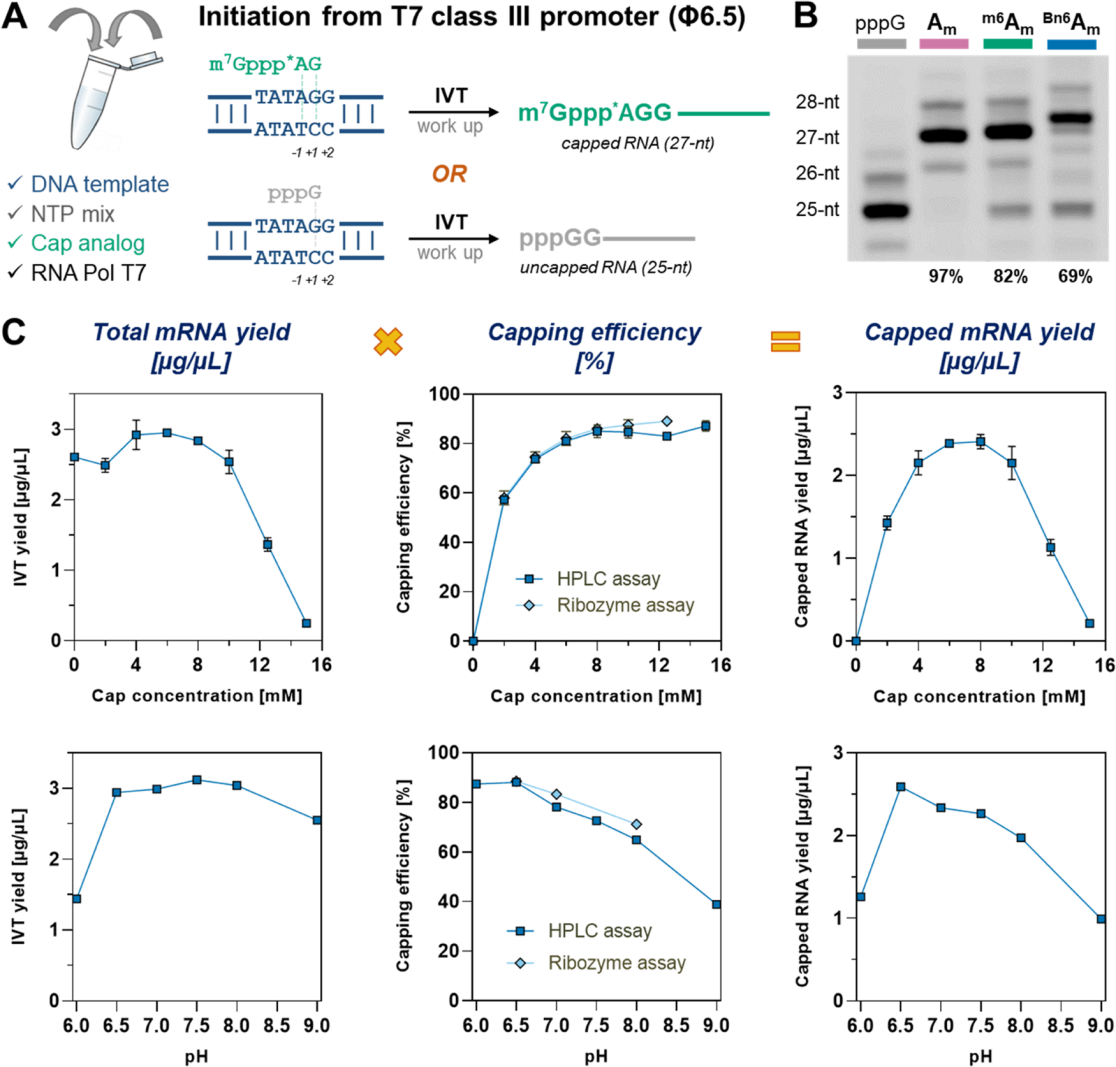
m^7^Gppp^Bn6^A_m_pG initiates transcription from the T7 class III promoter (φ6.5) and GGG transcription start site. (A) Possible scenarios for the initiation of transcription occurring in the presence of *AG-type trinucleotides (such as m^7^GpppApG) and the studied DNA template; *A denotes *N*6-modified adenosine residue. (B) Capping efficiencies for short RNAs determined by gel electrophoresis. IVT reactions were performed in the presence of 1.25 μM template, 3 mM ATP, CTP, UTP, and 0.75 mM GTP, 6 mM cap analogue, at pH 7.9 (for details, see the Experimental Section). (C) Capping efficiencies (determined by two methods) and IVT yields (determined spectrophotometrically after initial purification) as a function of *AvantCap* concentration and pH. IVT reactions were performed in the presence of 25 mM MgCl_2_, 40 ng/μL template, 5 mM ATP, CTP, UTP, 4 mM GTP, and 10 mM cap analogue (for optimizing pH) or various cap concentrations at pH 6.5.

We have recently shown that *N*6-methylation of adenosine does not substantially impair the ability of trinucleotide cap analogues to prime in vitro transcription reaction (up to 80% of capping efficiency on short 35-nt RNAs obtained with m^7^Gppp^m6^A_m_pG versus 90% for those obtained with m^7^GpppA_m_pG).^21^ To assess how well is the more bulky *N*6-benzyl substituent accommodated by the T7 RNA polymerase, we performed an in vitro transcription (IVT) from DNA template containing a T7 class III promoter (Φ6.5) sequence (Figure 2A) and coding for short RNAs under generic IVT conditions. The transcripts were trimmed by DNAzyme to reduce the heterogeneity of their 3′ ends,^38^ purified, and analyzed by gel electrophoresis to assess the ratio of capped and uncapped RNAs (capping efficiency; for details, see the Experimental Section). We found that the capping efficiency negatively correlates with the size of the *N*6-adenine substituent, but nonetheless, m^7^Gppp^Bn6^A_m_pG was incorporated into 69% of the transcribed RNAs, compared to 97% for m^7^GpppA_m_pG and 82% for m^7^Gppp^m6^A_m_pG under the same conditions (Figure 2B). These observations can be explained by the fact that in order to pair with the thymidine of the DNA template, both ^m6^A_m_ and ^Bn6^A_m_ have to adopt the unfavorable anti-conformation of the *N*6 substituent,^39^ which reduces the annealing rate.^40^

We then moved on to full-length mRNA (∼1000-nt) and attempted to optimize the IVT conditions to maximize both the capping efficiency and RNA yield. Extensive optimization of the IVT reaction mix in terms of component concentration and buffer composition revealed that cap concentration, pH of the buffer, and magnesium ion concentration are the most crucial factors affecting both capping efficiency and IVT yield (Figures 2C and S1). Capping efficiencies reaching 90% were achieved under the optimized conditions (8–10 mM cap, pH 6.5, and 25 mM MgCl_2_) for model mRNA. The conditions provided IVT yields of 2.5–4.3 mg/mL and capping efficiencies from 80 to 90% for most of the studied mRNAs (Table S2).

**Figure 3:**
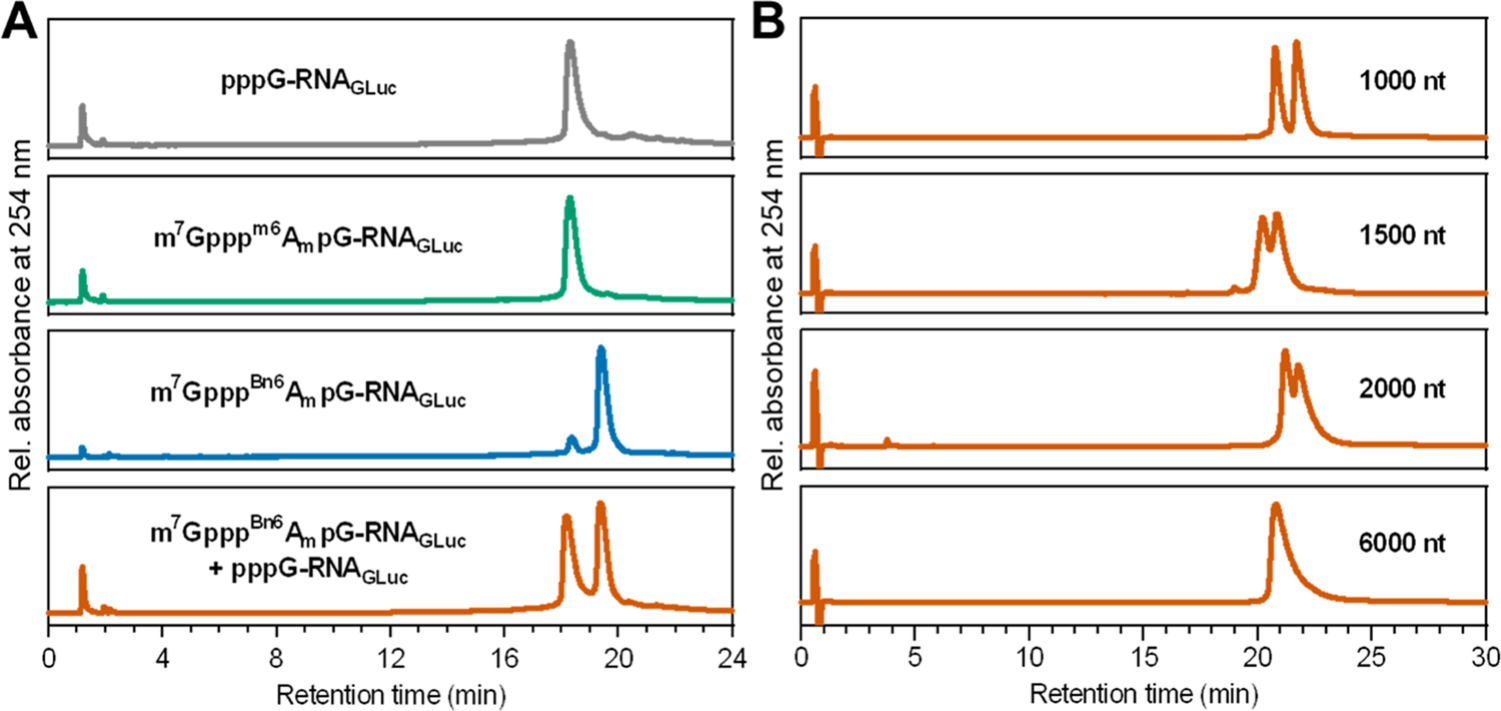
*N*6-Benzyl-2′-*O*-methyladenosine (^Bn6^A_m_) within the 5′ cap acts as an mRNA purification handle. (A) RP-HPLC analysis of *Gaussia* luciferase (Gluc) mRNA (956 nt) obtained by IVT carrying various 5′ terminal structures: uncapped mRNA (gray), m^7^Gppp^m6^A_m_pG-capped mRNA (green), m^7^Gppp^Bn6^A_m_pG-capped mRNA (blue), and 1:1 mixture of m^7^Gppp^Bn6^A_m_pG-capped mRNA and pppG-mRNA (orange), revealing that the presence of ^Bn6^A_m_ delays mRNA retention and enables the separation of capped mRNA (*R*_t_ = 19.4 min) and uncapped mRNA (*R*_t_ = 18.2 min). RP-HPLC conditions are given in the Experimental section. (B) RP-HPLC analyses of ∼1:1 mixtures of m^7^Gppp^Bn6^A_m_pG-capped transcripts of different lengths (1000, 1500, 2000, and 6000 nt) and corresponding uncapped mRNAs. RP-HPLC conditions are given in the Experimental Section.

When analyzing RNA integrity by HPLC, we surprisingly found that m^7^Gppp^Bn6^A_m_pG-capped mRNA had significantly longer retention time than the corresponding uncapped mRNA or mRNA carrying m^7^GpppA_m_pG or m^7^Gppp^m6^A_m_pG at the 5′ end (Figure 3A). This “hydrophobic effect” caused by the presence of the benzyl group in mRNA was observed for different mRNAs up to 2000 nt in length, in each case allowing straightforward separation of capped and uncapped (5′-triphosphorylated) RNAs (Figure 3B). A similar effect was observed before but for much bulkier substituents, such as fluorescent tags^41^ or photocleavable groups.^42^ This useful phenomenon can be harnessed for the RP-HPLC purification of mRNAs and for direct assessment of capping efficiency without the typical processing of the mRNA sample, which relies on cleaving the 5′-terminal sequence by an enzyme, DNAzyme, or ribozyme followed by electrophoretic analysis. The capping efficiency values obtained by the traditional ribozyme-based assay are in excellent agreement with values determined by the direct analysis of a small aliquot (0.5–1 μg) of mRNA by RP-HPLC (Figure 2C).

Additionally, we have noticed that initially purified mRNAs cotranscriptionally capped with *AvantCap* contain a reduced amount of dsRNA impurities in comparison to analogous transcripts with m^7^GpppA_m_pG (Figure S2). This feature was observed regardless of the applied purification method (affinity chromatography by oligo(dT)_25_ resin or purification using cellulose) or mRNA length and sequence composition. Therefore, we conclude that *AvantCap* can be harnessed to produce mRNAs with a low dsRNA content.

#### ^Bn6^A_m_ at the Transcription Start Site Increases mRNA Translation in Certain Cell Lines

**Figure 4:**
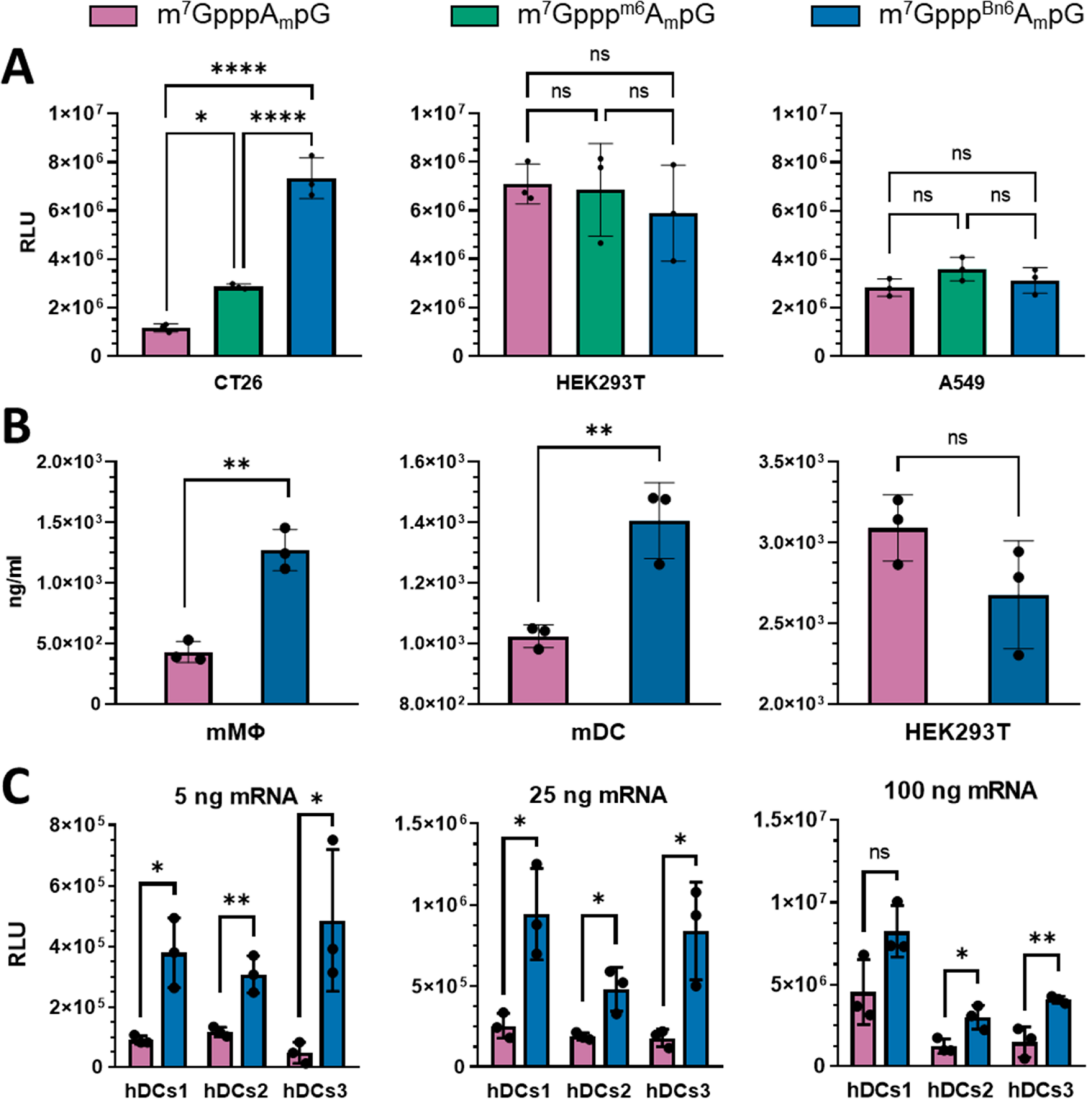
*N*6-Benzyladenosine within the mRNA 5′ cap increases protein production in certain mammalian cells. (A) Firefly luciferase (Fluc)-dependent luminescence of murine colon carcinoma (CT26, 10^4^ cells per well), human embryonic kidney (HEK293T, 10^4^ cells per well) and human lung carcinoma (A549, 10 ^4^ cells per well) cells 6 h post transfection with 50 ng of Fluc mRNA; data show relative luminescence units (RLUs) means ± SD, *n* = 3, *P < 0.05, ****P < 0.001, ns—not significant, one-way ANOVA with Tukey’s multiple comparison test. (B) Human erythropoietin (hEPO) concentration in the culture medium of primary murine bone marrow (BM)-derived macrophages (mMΦ, 5 × 10^4^ cells per well), murine BM-derived dendritic cells (mDC, 5 × 10^4^ cells), and human embryonic kidney (HEK293T, 10^4^ cells per well) cells 24 h post transfection with 200 ng of hEPO mRNA; data show hEPO concentrations (ng/mL) with means ± SD, *n* = 3, **P < 0.01, ns—not significant, two-tailed unpaired *t* test. (C) *Gaussia* luciferase (Gluc)-dependent luminescence of human monocyte-derived dendritic cells (hDCs, 5 × 10^4^ cells per well) differentiated from monocytes of three healthy adult males, transfected with 5, 25, or 100 ng of Gluc mRNA; data show total protein production (sum of relative luminescence units [RLUs] in daily measures from day 1 until 6 post transfection) with means ± SD, *n* = 3, * *P* < 0.05, **P < 0.01, ns—not significant, two-tailed unpaired *t* test.

After optimizing the IVT and purification protocols, we prepared a series of mRNAs encoding different reporter proteins (Tables S1 and S2) and tested them for translational activity in various cultured cell lines to gain first insights into the biological activity of mRNAs carrying *AvantCap*. All mRNAs were purified by affinity chromatography followed by RP-HPLC, and their purities, capping efficiencies, and homogeneities were verified (Table S2). Firefly luciferase (Fluc)-encoding mRNAs capped with m^7^Gppp^Bn6^A_m_pG, m^7^Gppp^m6^A_m_pG, or m^7^GpppA_m_pG (Table S2) were transfected into colorectal cancer (CT26), human lung carcinoma (A549), and human kidney embryonic (HEK293T) cells, and the luminescence dependent on the Fluc protein production was determined (Figure 4A). We found that the translational properties of mRNAs were cell culture-dependent. In CT26 cells, protein production from mRNAs capped with m^7^Gppp^Bn6^A_m_pG was higher than those capped with m^7^Gppp^m6^A_m_pG and m^7^GpppA_m_pG, while in HEK293T and A549 cells, the protein levels were comparable. Next, we prepared a series of human erythropoietin (hEPO) encoding mRNAs capped with m^7^Gppp^Bn6^A_m_pG or m^7^GpppA_m_pG (Table S2) and transfected them into primary bone marrow (BM)-derived murine macrophages, primary bone marrow (BM)-derived dendritic cells, or HEK293T cells. We observed increased protein production in both primary murine cells and HEK293T cells (Figure 4B). Finally, we also prepared *Gaussia* luciferase (Gluc) encoding mRNAs capped with either m^7^Gppp^Bn6^A_m_pG or m^7^GpppA_m_pG, and we compared their translation at different mRNA doses in human dendritic cells differentiated from monocytes of three healthy adult males (Figures 4C and S5). In each case, we observed 2- to 10-fold higher expression of the reporter protein from mRNAs capped with m^7^Gppp^Bn6^A_m_pG.

#### ^Bn6^A_m_ at the Transcription Start Site Increases mRNA Translation In Vivo in Mice

**Figure 5:**
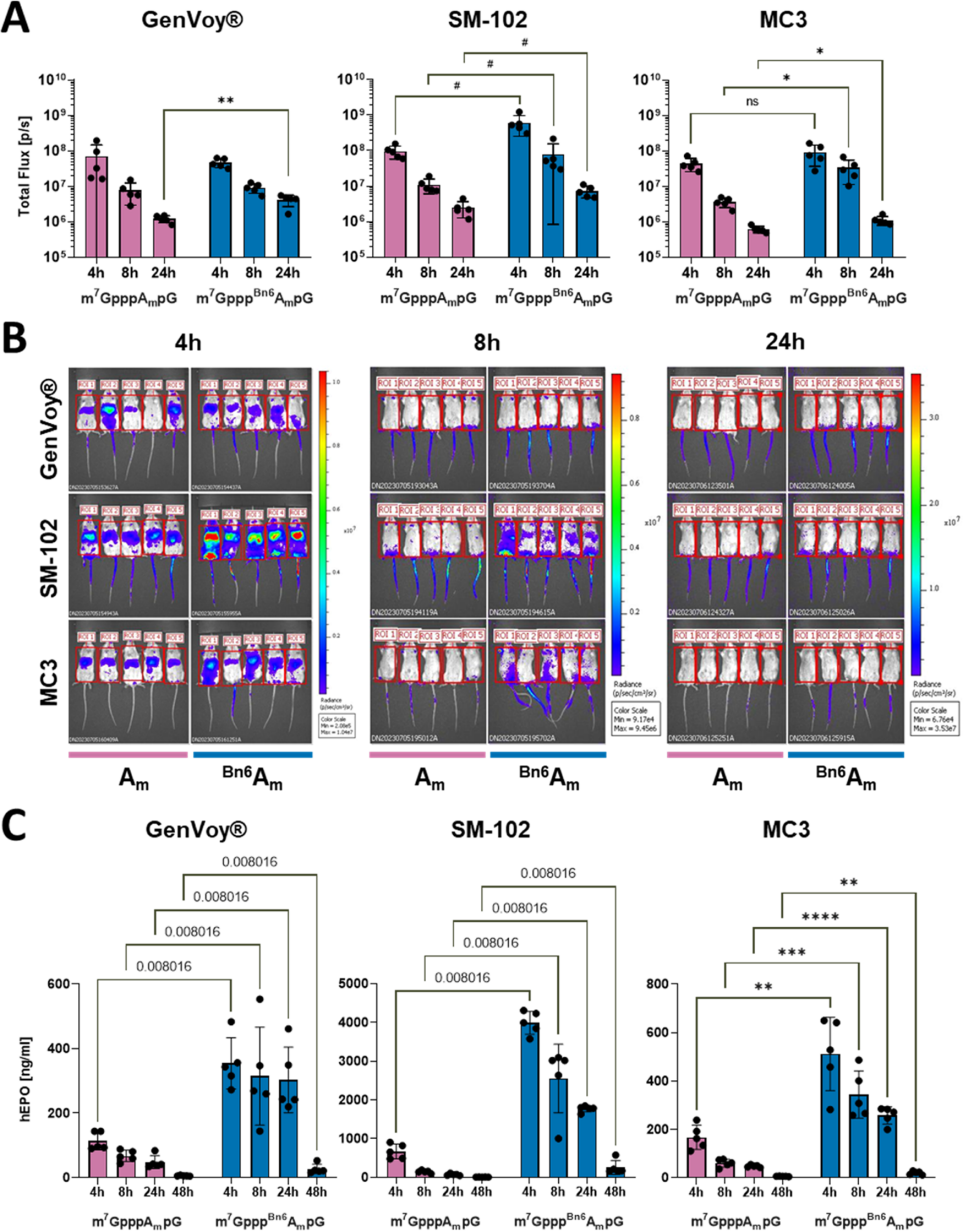
mRNA capped with m^7^Gppp^Bn6^A_m_pG (*AvantCap*) yields superior protein production in vivo. (A) Intravital bioluminescence at 4, 8, and 24 h in BALB/c mice injected intravenously (i.v.) with firefly luciferase (Fluc)-encoding mRNAs, capped with m^7^GpppA_m_pG or m^7^Gppp^Bn6^A_m_pG, and formulated using different ionizable lipids (GenVoy-ILM, SM-102, and MC3). Flux [p/s] mean values ± SD, *n* = 5, GenVoy-ILM and MC3 data: *P < 0.05, **P < 0.01, multiple unpaired *t* test; SM-102 data: Mann–Whitney test, *q* values [false discovery rate (FDR)-adjusted *P-*values] are shown. Note the logarithmic scale on the *y*-axis. (B) Raw bioluminescence images of mice inoculated with Fluc encoding mRNA (scale for bioluminescent signals at the right). (C) Human erythropoietin (hEPO) serum concentrations 4, 8, 24, and 48 h in C57BL/6 mice injected i.v. with hEPO encoding mRNAs, capped with m^7^GpppA_m_pG or m^7^Gppp^Bn6^A_m_pG, and formulated using different ionizable lipids (GenVoy-ILM, SM-102, and MC3). Data show mean values ± SD, *n* = 5, GenVoy-ILM and SM-102 data: Mann–Whitney test, *q* values (FDR-adjusted P-values) are shown; MC3 data: *P < 0.05, **P < 0.01, ***P < 0.001, ****P < 0.0001 multiple unpaired *t* test.

Next, we investigated how the presence of benzyl modification affects mRNA expression in vivo. First, we used the wild-type Fluc reporter mRNAs capped with m^7^GpppA_m_pG or m^7^Gppp^Bn6^A_m_pG. mRNA purity and integrity were confirmed before each experiment (Figure S3 and Table S2). Then, mRNAs were formulated into lipid nanoparticles (LNPs) using various ionizable lipids (GeneVoy-ILM, SM-102, or MC3) or complexed with commercially available transfection reagent (TransIT) and administered intravenously (iv) into mice. The luciferase activity was determined at multiple time points (Figures 5A,B and S6). The absolute and relative activities of differently capped mRNAs depended on the formulation type. The highest expression of a reporter gene was observed in mice treated with mRNAs formulated in SM-102 lipid, which also showed notable difference between m^7^GpppA_m_pG and m^7^Gppp^Bn6^A_m_pG-capped mRNAs (over a 6-fold higher expression from ^Bn6^A_m_ RNA at 4 and 8 h time points and almost 3-fold at 24 h; Figure 5). Significantly higher expression was also observed when mRNAs were delivered with TransIT (Figure S6), while for other formulations, the differences were statistically significant for selected time points only (Figure 5). We then used a different reporter protein that can be quantified using ELISA. To that end, we compared protein outputs from mRNAs encoding hEPO as a model of a therapeutically relevant protein (Figure 5C). Again, purified mRNAs (Figure S4) formulated with SM-102 lipid provided the highest expression, but in this case, the advantage of *AvantCap* over unmodified cap-1 was evident in all formulations at all time points. At 4 h post injection of SM-102 LNPs, the concentration of hEPO in mice blood serum was 6-fold higher in mice treated with ^Bn6^A_m_-capped mRNA than in those treated with m^7^GpppA_m_pG-RNA, and the ratio increased over time. Using a different potentially therapeutic mRNA, we detected over 5-fold higher α1-antitrypsin (hA1AT) concentrations in the sera of mice inoculated i.v. with TransIT-formulated hA1AT-encoding mRNAs capped with m^7^Gppp^Bn6^A_m_pG as compared to m^7^GpppA_m_pG-capped mRNA (Figure S7D). hA1AT levels were also increased when HEK293T and A549 cells were transfected with mRNA capped with m^7^Gppp^Bn6^A_m_pG compared to that of m^7^GpppA_m_pG-capped mRNA (Figure S7A–C).

#### m^7^Gppp^Bn6^A_m_pG-Capped mRNA Shows Superior Therapeutic Activity in Cancer Models

**Figure 6:**
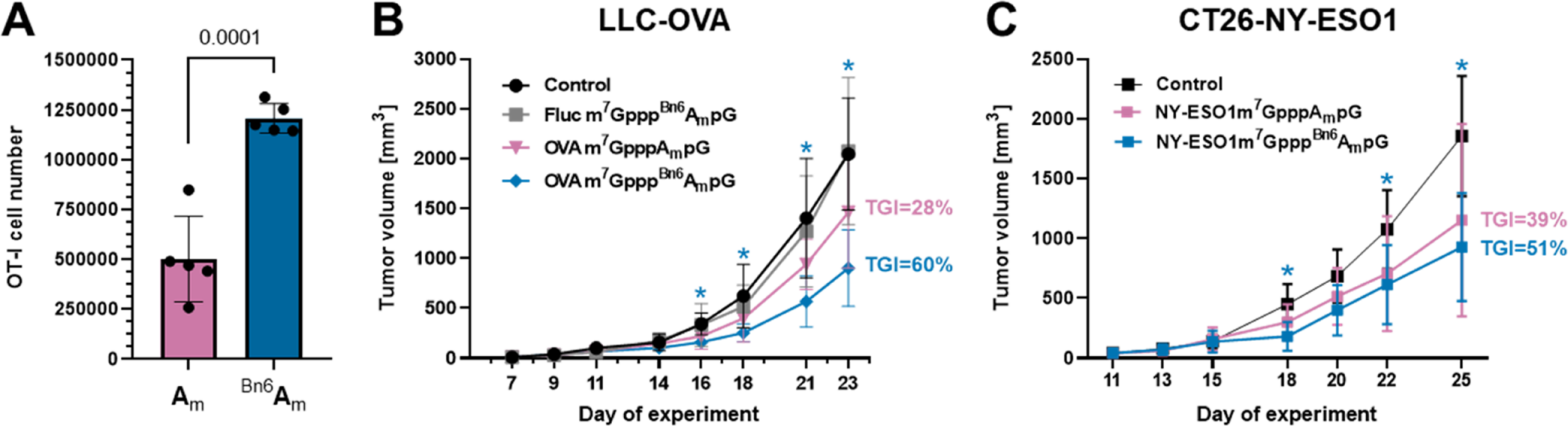
mRNA capped with m^7^Gppp^Bn6^A_m_pG (*AvantCap*) shows therapeutic activity in cancer models. (A) Quantitative OT-I T-cell proliferation in response to in vivo delivery of SIINFEKL antigen/peptide-encoding m^7^GpppA_m_pG or m^7^Gppp^Bn6^A_m_pG-capped mRNA. Mice were administered intravenously (i.v.) with 7.5 ng of mRNA in TransIT formulation. T-cell numbers were normalized to CountBright Absolute Counting Beads (ThermoFisher Scientific). Data show OT-I T-cell cell numbers in the spleen after 72 h of proliferation in vivo; data show means ± SD, *n* = 5, two-tailed unpaired *t* test. (B) C57BL/6 mice were inoculated with Lewis lung carcinoma (LLC) cells stably expressing a model antigen–ovalbumin (OVA) and weekly treated i.v. with 100 ng of OVA-encoding m^7^GpppA_m_pG or m^7^Gppp^Bn6^A_m_pG-capped mRNA formulated in TransIT. Tumor volumes are shown as means ± SD, *n* = 8. Mixed-effect analysis with Dunnett’s multiple comparison test, m^7^Gppp^Bn6^A_m_pG-capped mRNA vs control: day 16: *P* = 0.0060; day 18: *P* = 0.0410; day 21: *P* = 0.0168; day 23: *P* = 0.0080. Control mice received PBS. m^7^Gppp^Bn6^A_m_pG-capped mRNA encoding human hEPO served as a tumor antigen-irrelevant control. TGI (tumor growth inhibition compared to controls). (C) BALB/c mice were inoculated with CT26 murine adenocarcinoma (CT26) cells stably expressing a human tumor antigen (NY-ESO1) and weekly treated i.v. with 100 ng of NY-ESO1-encoding m^7^GpppA_m_pG or m^7^Gppp^Bn6^A_m_pG-capped mRNA formulated in TransIT. Tumor volumes are shown as means ± SD, *n* = 8. 2-way ANOVA with Dunnett’s multiple comparison test, m^7^Gppp^Bn6^A_m_pG-capped mRNA vs control: day 18: *P* = 0.0079; day 22: *P* = 0.0360; day 25: *P* = 0.0046. Control mice received PBS. TGI—tumor growth inhibition compared to controls.

To verify if mRNAs capped with m^7^Gppp^Bn6^A_m_pG can exert therapeutic effects, we carried out in vivo experiments in mice using two different therapeutically relevant mRNAs. First, we used mRNAs encoding cancer-associated antigens, a strategy used in anticancer vaccinations.^43^ Intravenous administration of mRNA encoding SIINFEKL peptide from ovalbumin (OVA) followed by the adoptive transfer of OVA-recognizing OT-I T-cells isolated from the spleens of transgenic C57BL/6-Tg(TcraTcrb)1100Mjb/J mice resulted in the significant expansion of antigen-specific T-cells in recipient animals (Figure S8). Notably, over 2-fold higher numbers of OT-I T-cells were observed in recipient mice when mRNA was capped with m^7^Gppp^Bn6^A_m_pG as compared to mice that received mRNA capped with m^7^GpppA_m_pG (Figure 6A). Encouraged by the results showing that mRNA encoding model antigen can induce the expansion of antigen-specific T-cells, we investigated antitumor effects of mRNA encoding tumor antigens in two different tumor models. Lewis lung carcinoma (LLC) cells were stably transduced with OVA and inoculated into C57BL/6 mice. Mice were then treated i.v. with 100 ng of mRNA encoding irrelevant protein (Fluc) or OVA on days 7, 14, and 21. Inhibition of tumor growth was observed only in mice that received OVA-encoding mRNA, and statistically significant (vs controls) inhibition was observed only in mice that received mRNA capped with m^7^Gppp^Bn6^A_m_pG (Figure 6B). Similarly, significant inhibition of tumor growth was observed in BALB/c mice inoculated with CT26 colon adenocarcinoma cells stably transduced with a model human antigen (NY-ESO1) and treated with mRNA encoding NY-ESO1 and capped with m^7^Gppp^Bn6^A_m_pG (Figure 6C). Although no formal toxicology studies were performed, we have not noticed any gross adverse effects of mRNA (capped with either m^7^Gppp^Bn6^A_m_pG or m^7^GpppA_m_pG) administration, such as weight loss, hunched posture, ruffled hair coat, lethargy, anorexia, diarrhea, or neurological impairment.

### Biochemical Consequences of Incorporating *AvantCap*

To gain deeper insight into the molecular mechanisms underlying the increased translation yield of mRNAs containing the ^Bn6^A_m_ cap, we performed a series of biochemical and biophysical assays to verify which mRNA-related processes are affected by the modification. We tested the susceptibility of *AvantCap* to dealkylation by FTO and its affinity to several members of the eIF4E (eukaryotic translation initiation factor 4E) family of proteins. The ^Bn6^A_m_-capped mRNAs were characterized for translation in a cell-free system and for susceptibility to decapping by Dcp2, which contributes to the overall mRNA stability.

#### ^Bn6^A_m_ Is Not a Substrate for FTO

**Figure 7:**
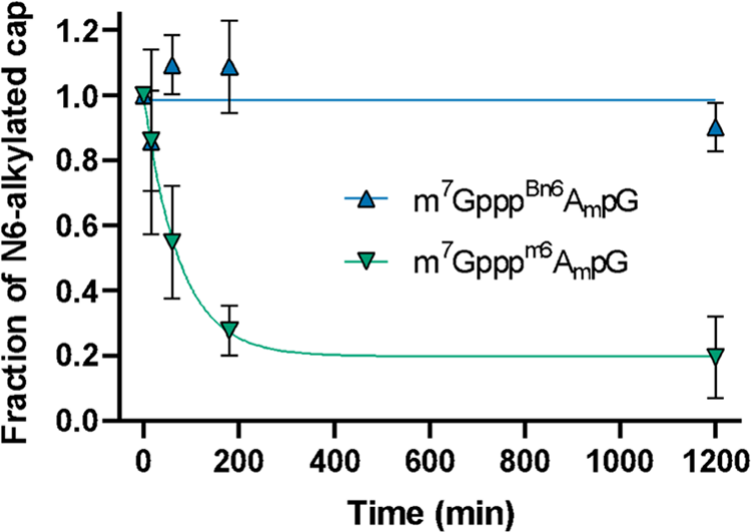
Cap structure containing ^Bn6^A_m_ is resistant to removal by FTO. ^Bn6^A_m_- or ^m6^A_m_-containing cap analogues were incubated with FTO, and the amount of *N*6-alkylated cap remaining in the mixture was assessed by RP-HPLC-MS. Reaction conditions: 20 μM cap and 2 μM FTO in 50 mM HEPES pH 7, containing 150 mM KCl, 75 μM Fe(II), 300 μM 2-oxoglutarate, 2 mM ascorbic acid. Similar data obtained for the 200 μM cap are shown in Figure S9.

Since the ^Bn6^A_m_ cap was designed as an ^m6^A_m_ cap analogue, we first tested its susceptibility to *N*6-dealkylation by FTO—the only known ^m6^A_m_ eraser—by monitoring the reaction progress by RP-HPLC with MS detection (Figure 7). While m^7^Gppp^m6^A_m_pG was demethylated with a half-life of about 50 min, only up to 10% of *AvantCap* was dealkylated even after 20 h of incubation with the enzyme. Also, using a 10-times higher concentration of the m^7^Gppp^Bn6^A_m_pG, we have not observed any significant dealkylation by FTO (Figure S9). The data suggest that the *AvantCap* can be considered as a stable analogue of ^m6^A_m_ cap under physiological conditions.

#### ^Bn6^A_m_ Has Minor Stabilizing Effect on the Interaction with Translation Initiation Factor (eIF4E) and Does Not Improve mRNA Translation in a Cell-Free System

**Figure 8:**
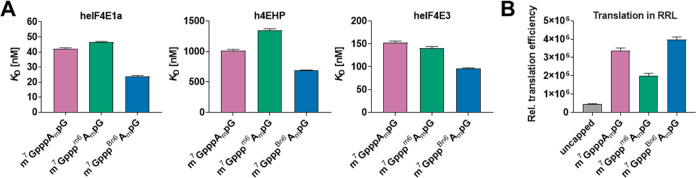
(A) Binding affinities of *AvantCap* (**1**) and reference compounds for human translation initiation factor 4E (eIF4E) isoforms 1a and 3 and human 4E homologous protein (4EHP). (B) Relative translation efficiencies of mRNA capped with m^7^Gppp^Bn6^A_m_pG (**1**) and reference analogues in the rabbit reticulocyte lysate (RRL).

The affinity of a cap analogue to eIF4E protein is often correlated with the translation efficiency of such capped mRNAs; therefore, we quantified the binding interactions between trinucleotide cap structures and eIF4E using time-synchronized fluorescence quenching titration (FQT). To consider the potential effects of ^Bn6^A_m_ modification on both translation initiation and its inhibition, we analyzed interactions with three members of the eIF4E family (Table 1 and Figure 8A): heIF4E1a, which is a part of the eIF4F complex responsible for ribosome recruitment, h4EHP, and heIF4E3, both of which lack the ability to bind eIF4G and thus act as translation suppressors. We reasoned that the increased translational capacity of mRNAs capped with m^7^Gppp^Bn6^A_m_pG may be explained by either stabilization of the interaction with eIF4E or destabilization of the interactions with h4EHP or eIF4E3. For all eIF4Es studied, we observed no significant effect for ^m6^A_m_ compared to A_m_, and for ^Bn6^A_m_, we observed only a minor (1.5–1.75-fold) increase in binding affinity.

**Table 1:** Binding Affinities of m^7^Gppp^Bn6^A_m_pG (**1**) and Reference Compounds for Human Translation Initiation Factor 4E (eIF4E) Isoforms 1a and 3 and Human 4E Homologous Protein (4EHP)

	heIF4E1a		h4EHP (eIF4E2)		heIF4E3	
cap analogues	*K*_D_ (nM)	*K*_D_/*K*_Dcap-1_	*K*_D_ (nM)	*K*_D_/*K*_Dcap-1_	*K*_D_ (nM)	*K*_D_/*K*_Dcap-1_
m^7^GTP	7.8 ± 0.3	0.19 ± 0.01	611 ± 19	0.60 ± 0.03	129 ± 14	0.84 ± 0.11
m^7^GpppA_m_pG	42.1 ± 0.7	1	1015 ± 19	1	153 ± 3	1
m^7^Gppp^m6^A_m_pG	46.4 ± 0.6	1.10 ± 0.03	1342 ± 32	1.32 ± 0.06	140 ± 4	0.92 ± 0.04
m^7^Gppp^Bn6^A_m_pG	23.9 ± 0.5	0.57 ± 0.02	687 ± 10	0.68 ± 0.02	95.7 ± 1.7	0.63 ± 0.02

We also compared the translation efficiencies of mRNAs capped with cap-1, ^m6^A_m_ cap, and *AvantCap* in nuclease-treated rabbit reticulocyte lysates (RRL, Figure 8B). Consistent with the previous report,^13^ we observed a 40% reduction in protein production from mRNA with the ^m6^A_m_ cap, which was completely reversed for the *N*6-benzyl modification. Still, this effect is unlikely to account for up to a 6-fold increase in translation yield observed in hDCs and in vivo.

#### AvantCap Is Susceptible to Decapping and Does Not Affect mRNA Stability in Cultured Cells

**Figure 9:**
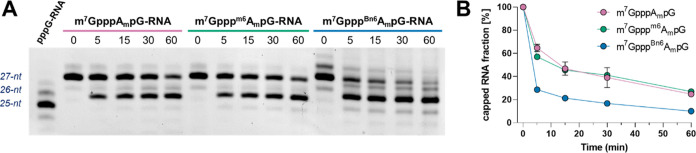
Susceptibility of short RNAs to decapping by the PNRC2-hDcp1/Dcp2 complex in vitro. Short 27-nt-capped RNAs (20 ng) were subjected to hDcp1/Dcp2 (13 nM) in complex with the regulatory peptide PNRC2^44^ for 60 min at 37 °C. Aliquots from different time points were resolved by polyacrylamide gel electrophoresis (PAGE), stained with SYBR gold and analyzed by densitometry. (A) Representative PAGE gel from single experiment and (B) results from triplicate experiments ± SEM. Error bars are not visible if smaller than data points. Individual data for all replicates are shown in Figure S11.

Methylation of the *N*6-position of cap-adjacent adenosine has been reported to interfere with decapping by Dcp2,^15^ but our previous in vitro studies suggested that the enzyme is insensitive to either 2′-*O* or *N*6-methylation of adenosine.^21^ We performed an in vitro decapping assay for short RNAs capped with m^7^Gppp^Bn6^A_m_pG, m^7^Gppp^m6^A_m_pG, and m^7^GpppA_m_pG and, surprisingly, found that RNAs carrying ^Bn6^A_m_ modification were slightly more susceptible to decapping compared to both A_m_- and ^m6^A_m_-capped transcripts (Figure 9). We have also observed no difference in mRNA stability in HEK293T cells electroporated with mRNA capped with m^7^Gppp^Bn6^A_m_pG, m^7^Gppp^m6^A_m_pG, or m^7^GpppA_m_pG as evidenced by RT-qPCR (Figure S10).

### Protein Fraction Pulled-Down from HEK293F Cell Extract Using Immobilized *AvantCap* Is Enriched in eIF4E and eIF3

**Figure 10:**
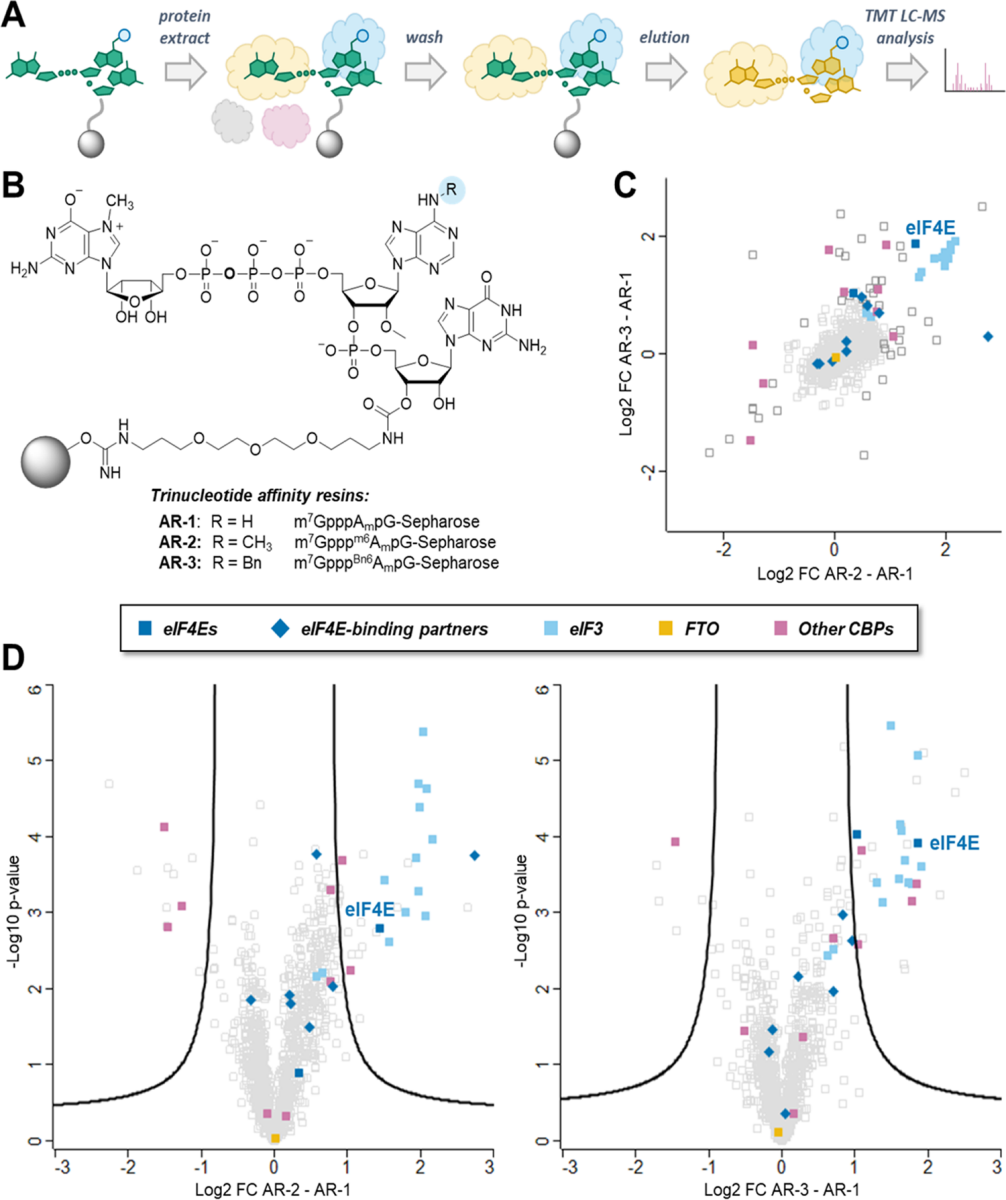
Pull-down assay with protein extract from HEK293F cells. (A) The workflow of the experiment. (B) Chemical structure of trinucleotide affinity resins. (C) Dot plot representations of correlation between log2 fold change **AR-3**/**AR-1** and log2 fold change **AR-2**/**AR-1**. (D) Volcano plots displaying the log2 fold change (log2 FC) against the *t* test-derived −log 10 statistical *p*-value (−log *p*-value) for the protein groups in the eluates from trinucleotide cap affinity resins **AR-2**/**AR-1** (*n* = 3) and **AR-2**/**AR-1** (*n* = 3). Student’s *t* tests (two-sided, unpaired) were performed to assess binding preferences of protein groups to **AR-2** and **AR-3** resins relative to **AR-1** resin.

Since none of the biochemical assays provided a satisfactory explanation for the mechanism underlying the increased translation of mRNAs capped with *AvantCap* in several cell lines and in vivo, we searched for the potential selective interactors of ^m6^A_m_ and ^Bn6^A_m_ caps in cell lysate using a pull-down assay (Figure 10A). To this end, we synthesized a series of trinucleotide cap analogues **2a**–**c** functionalized with an amine-terminated linker at the guanosine ribose and immobilized them on BrCN-activated Sepharose (Figure S12A). The resulting affinity resins **AR-1** (A_m_), **AR-2** (^m6^A_m_), and **AR-3** (^Bn6^A_m_; Figure 10B) were incubated with the HEK293F cell extract in the presence of GTP to limit nonspecific interactions. The pulled-down proteins were eluted with the corresponding trinucleotide cap analogue (m^7^GpppA_m_pG for **AR-1**, m^7^Gppp^m6^A_m_pG for **AR-2**, and m^7^Gppp^Bn6^A_m_pG for **AR-3**), digested with trypsin, labeled with isobaric tags (TMT), and analyzed by shotgun proteomics. The list of identified proteins and the results of Student’s *t* tests (two-sided, unpaired) performed to assess binding preferences of proteins to the resins are given in Table S3. Based on the statistical method applied, 29 protein groups were classified as preferentially binding to **AR-2** over **AR-1** and 36 as preferentially binding to **AR-3** over **AR-1** (Figure 10D); 17 of these were enriched in both **AR-2** and **AR-3** over **AR-1** (Figure 10C). Notably, the common group of proteins contained eIF4E, DcpS, and eIF3 (11 of 13 subunits; the remaining two—eIF3J and eIF3K—were also enriched but did not pass our stringent statistical significance test), which were rather abundant in these eluates, as estimated by their iBAQ values (Figure S12B).^45^ The remaining four are present at lower levels and include RPA1, DNAJC10, R3HCC1L, and UPP1, none of which is directly related to mRNA translation. We also found 7 proteins whose concentrations were significantly reduced in both **AR-2** and **AR-3** eluates as compared to that of **AR-1**. These included NCBP1, which is known to stabilize the interaction of the cap with NCBP2, and several other nuclear RNA-binding proteins: FUS, TIA1, TAF15, EWSR1, HNRNPH3, and HNRNPA2B1. Consistent with a previous report on snRNA,^14^^m6^A_m_ (and ^Bn6^A_m_ but to a lesser extent) appeared to destabilize the interactions of cap with NCBP2. We observed no differences in the abundance of FTO among the samples, although it was generally present at low levels (Figure S12B).

One of the cap-dependent pathways of translation initiation in human cells relies on cap-binding activity of eIF3d, a subunit of the 800-kilodalton eIF3 complex. Based on the results of pull-down assay, we hypothesized that the increased mRNA translation might be caused by the preferential engagement of alternative cap-dependent translation initiation that relies on the cap-binding activity of eIF3d.^46^ To verify this hypothesis, we preincubated hDCs with an mTOR inhibitor, INK128, which leads to dephosphorylation of the 4E-BP1, allowing its binding to and inhibiting eIF4E, thereby creating conditions for alternative cap-dependent translation initiation.^47−49^ Transfection of hDCs with mRNA encoding hEPO led to higher protein concentrations when mRNA was capped with m^7^Gppp^Bn6^A_m_pG (Figure S13A, left). Preincubation with INK128 strongly attenuated mRNA translation for all tested mRNAs, but this effect was more pronounced when mRNA was capped with m^7^Gppp^m6^A_m_pG or m^7^GpppA_m_pG as compared with m^7^Gppp^Bn6^A_m_pG (Figure S13A, right). eIF3d makes specific contacts with the cap, and these interactions are essential for the assembly of translation initiation complexes on eIF3-specialized mRNAs such as the one for the cell proliferation regulator c-JUN.^47^ c-JUN mRNA contains an inhibitory RNA element that blocks eIF4E recruitment, thus enforcing alternative cap recognition by eIF3d. We have prepared mRNAs encoding Fluc with variants of c-JUN 5′UTRs: (i) C-JUN—300 nt of the wild-type c-JUN and (ii) ΔeIF3—C-JUN 5′UTR without the stem loop responsible for eIF3 binding (nucleotide positions 181–214) (Figure S13B). Transfection of these mRNA constructs into hDCs revealed that while the luminescence with normal 5′-UTRs (human β globin) is 20% higher when mRNA is capped with m^7^Gppp^Bn6^A_m_pG (as compared with mRNA capped with m^7^Gppp^m6^A_m_pG), the difference is almost 4-fold higher when C-JUN is used as 5′-UTR (Figure S13C). Deletion of the stem loop responsible for eIF3 binding abrogates the translational advantage of mRNA capped with m^7^Gppp^Bn6^A_m_pG (Figure S13C). These results suggest that the advantage of using m^7^Gppp^Bn6^A_m_pG may be at least to some extent due to the eIF3-dependent translation initiation and interaction of eIF3d with *AvantCap*, although this requires further experimental verification.

## Discussion

The success of mRNA-based therapeutic approaches has been facilitated in large part by the use of chemical modifications to manipulate the biological properties. The prime example is the substitution of uridines with (1-methyl)pseudouridines in the RNA body to reduce the undesired reactogenicity of mRNA,^50^ but some chemical modifications of mRNA ends have also proven to be beneficial in this context.^32^ Here, we report *AvantCap*—a novel synthetically easily accessible trinucleotide cap analogue containing an *N*6-benzyl-2′-*O*-methyladenylate moiety (^Bn^^6^A_m_) that confers superior properties to in vitro-transcribed capped mRNAs with potential benefits for mRNA therapeutics (Figure 1 and Scheme 1). The design of this analogue was inspired by the naturally occurring 5′-terminal ^m6^A_m_ mark present in many endogenous mammalian mRNAs of yet not fully understood function. Here, by replacing the *N*6-methyl in m^7^Gppp^m6^A_m_ with a benzyl group, we obtained a mimic that is more hydrophobic, resistant to demethylation by FTO, efficiently incorporated into the RNA 5′ end, and yields mRNAs that are more translationally active in many of the tested biological settings (Figures 2 and 7). The presence of the ^Bn6^A_m_ modification results in a hydrophobic effect,^41^ which is useful for RNA purification on RP-HPLC columns as it significantly increases RNA retention (Figures 2 and 3). For RNAs up to 2000 nt, this effect is strong enough to allow the separation of capped and uncapped RNAs, even on a preparative scale. We speculate that even for longer RNAs, for which no clear separation is observed, the enrichment of capped RNA species is possible. In addition, mRNAs produced in the presence of *AvantCap* contain less dsRNA impurities than mRNAs obtained in the presence of m^7^GpppA_m_pG (Figure S2). The purity of mRNAs, including dsRNA and uncapped RNA contents, has recently been identified as one of the most important factors for their therapeutic applications, particularly in anticancer and protein replacement approaches.^51^ Therefore, we believe that *AvantCap* can provide significant improvements in this area.

We investigated the biological properties of mRNAs capped with *AvantCap* in various biological settings, as recent studies have revealed that the translational properties of IVT-transcribed mRNAs may be cell-type-dependent. Indeed, we have found that in several mammalian cell lines such as HEK293 or A549, mRNAs carrying *AvantCap* have comparable properties to those of mRNAs carrying unmodified cap-1 (Figure 4). However, in other cell types such as CT26, macrophages, or dendritic cells, mRNAs carrying the ^Bn6^A_m_ mark afford significantly higher protein outputs. This effect is also observed in vivo for all reporter proteins tested, albeit its magnitude is varying depending on the mRNA and formulation type (Figure 5). Among three different LNP types, the formulation based on lipid SM-102 provided the best differentiation between differently capped mRNAs and showed up to a 6-fold higher expression of mRNAs carrying *AvantCap*, compared to mRNAs carrying the cap-1 structure. Even more pronounced superior activity of mRNAs carrying *AvantCap* was observed for the cationic-lipid transfection reagent (TransIT) (Figure S6). It is unclear why different lipid formulations result in such differences in the biological activity of mRNA, but it may be related to different cellular signaling pathways being activated by different delivery methods and/or different cell types being targeted in vivo. We also show that mRNAs carrying *AvantCap* show superior activity in therapeutic models of anticancer vaccines (Figures 6 and S8).

Trying to uncover the rationale behind the biological effects of *AvantCap*, we thoroughly characterized its biochemical properties in vitro. To that end, we analyzed binding affinity for eIF4E and translational properties in a cell-free system (Table 1 and Figure 8), susceptibility to enzymatic decapping (Figure 9), and dealkylation by FTO (Figure 7). These data indicate that m^7^Gppp^Bn6^A_m_pG is an FTO-resistant but not Dcp2-resistant mimic of m^7^Gppp^m6^A_m_pG, with slightly increased affinity to eIF4E. To further compare the biochemical properties of m^7^Gppp^Bn6^A_m_pG, m^7^Gppp^m6^A_m_pG, and m^7^GpppA_m_pG, we synthesized a series of trinucleotide affinity resins and performed a pull-down assay with cellular protein extracts (Figure 10). Two possible mechanisms emerge from this assay: (i) direct involvement of eIF3 in the differentiation between A_m_ and ^m6^A_m_/^Bn6^A_m_ caps or (ii) enhancement of selectivity toward eIF4E in a complex cellular environment upon *N*6 modification of the cap by reducing its affinity for off-target proteins. The first hypothesis seems attractive in the context of recent reports on alternative cap-dependent translation initiation using the eIF4G2 (DAP5)/eIF3d pathway activated under stress conditions.^49^ Cell culture experiments under conditions prohibitory to conventional translation pathway did not exclude the possibility of participation of *AvantCapped*-mRNAs in alternative translation mechanisms, but it requires further investigation. The second possibility is analogous to the mechanism of evading the innate immune response by forming the cap-1 structure, which is not recognized by IFIT proteins or RIG-I-like receptors. It is also possible that the observed differences in biological activity arise from subtly different impurity profiles for differently capped mRNAs that differentially activate cellular signaling pathways and the innate immune system. However, we have put significant effort into preparing high-quality mRNA by employing a two-step purification process, including RP-HPLC purification, and verifying the quality of the final mRNA products (Table S2 and Figures S3 and S4). Overall, the superior biological activity of *AvantCap* combined with the straightforward and scalable synthesis makes this analogue an attractive opportunity for advanced applications of IVT mRNA.

## Supporting Information Available

Experimental procedures and product characterization data, including NMR and HRMS spectra for new compounds (PDF)
Quality parameters of mRNAs prepared for experiments; capping efficiencies (determined by two methods) and IVT yields (determined spectrophotometrically for the samples purified using oligo(dT)_25_ resin) as a function of pH in the range of 6.0–7.0; representative quality control of the final samples of mRNA encoding firefly luciferase (Fluc) after whole purification process; time-dependent expression profiles of differently capped Gluc mRNAs in human dendritic cells; flow cytometry gating strategy for the identification of proliferating OT-I T-cells in the spleen; and pull-down assay with protein extract from HEK293F cells (PDF)
List of identified proteins and the results of Student’s *t* tests (two-sided, unpaired) performed to assess binding preferences of proteins to the resins (XLSX)
